# Assessment of Hearing and Vestibular Functions in a Post-COVID-19 Patient: A Clinical Case Study

**DOI:** 10.3390/diagnostics13010122

**Published:** 2022-12-30

**Authors:** Irina Germanovna Andreeva, Alisa Gvozdeva, Vera Pimenova, Varvara Ryabkova, Maria Lukashenko, Evelina Kamaeva, Valeria Shapkina, Lidia Soprun, Natalia Gavrilova, Tamara Viktorovna Fedotkina, Leonid Pavlovich Churilov, Yehuda Shoenfeld

**Affiliations:** 1 Sechenov Institute of Evolutionary Physiology and Biochemistry of the Russian Academy of Sciences (IEPhB RAS), Laboratory of Comparative Sensory Physiology, 194223 St. Petersburg, Russia; 2 Department of Therapy, Pavlov First St. Petersburg State Medical University, 197022 St. Petersburg, Russia; 3 Laboratory of the Mosaic of Autoimmunity, Saint-Petersburg State University, 199034 St. Petersburg, Russia; 4 Department of Hospital Surgery, Saint-Petersburg State University, 199034 St. Petersburg, Russia; 5 Department of Healthcare and Medical law, Saint-Petersburg State University, 199034 St. Petersburg, Russia; 6 Outpatient Clinic No. 1, Saint-Petersburg State University Clinic, 190103 St. Petersburg, Russia; 7 Department of Faculty Therapy, Saint-Petersburg State University, 199034 St. Petersburg, Russia; 8 St. Petersburg Research Institute of Phthisiopulmonology of the Ministry of Health of the Russian Federation, 191036 St. Petersburg, Russia; 9 Department of Pathology, Saint-Petersburg State University, 199034 St. Petersburg, Russia; 10 Zabludowicz Center for Autoimmune Diseases, Sheba Medical Center, Tel HaShomer 5265601, Israel; 11 Sackler Faculty of Medicine, Ariel University, Ariel 40700, Israel

**Keywords:** post-COVID-19 syndrome, autoimmunity, hearing loss, hearing tests, vestibular tests, gap detection test, dichotic test

## Abstract

SARS-CoV-2 infection may cause such complications as post-COVID-19 syndrome, which includes chronic fatigue, myalgia, arthralgia, as well as a variety of neurological manifestations, e.g., neuropathy of small fibers, hearing and vestibular dysfunction, and cognitive impairment. This clinical case describes a 41-year-old patient suffering from post-COVID-19 syndrome and chronic fatigue syndrome. A detailed examination was performed, including an in-depth study of peripheral and central hearing and vestibular functions, as well as small nerve fibers length and density in the skin and cornea of the eye. Contrary to expectations, no peripheral nervous system dysfunction was detected, despite the presence of dizziness and gait instability in the patient. Hearing tests (gap detection test and dichotic test) showed central auditory processing disorders. The evaluated lesion in the processing of temporal and verbal auditory information can be a significant factor contributing to additional overload of the neural activity and leading to chronic fatigue when performing daily activities in patients with CFS and post-COVID-19 complications.

## 1. Introduction

In 2020, the world faced a pandemic of the COVID-19 infection, which not only led to a dramatic increase in the number of patients suffering from viral pneumonia and respiratory failure, but also to the evaluation of a significant number of complications, known as post-covid syndrome [1,2]. In 2021, the World Health Organization introduced the Delphi consensus, where the criteria for this syndrome were described, allowing medical specialists to work with “post-COVID-19 condition” (U09.9) as a diagnosis [3]. The criteria for this syndrome specified the presence of symptoms for at least 2 months after the onset of the disease. The typical complaints included, among others, chronic fatigue, subfebrile hyperthermia, cognitive impairment, arthralgia, and myalgia. Autonomic nervous system dysfunction was also frequently described. [1,2,3,4].

Many symptoms of post-COVID-19 syndrome reminded medical specialists of those in chronic fatigue syndrome (CFS/ME) [5,6]. CFS/ME is a disease of unknown etiology, characterized by an increase of physical and mental fatigue, which persists after rest and is accompanied by a variety of autonomic symptoms [7]. Up to 70% of CFS patients note the presence of infectious triggers, predominantly viral etiology, associated with SARS-CoV-2 and Herpesviridae spp., followed by the progression of immunologic and autonomic complications [8,9]. In the pathogenesis of CFS/ME, neuroimmune, endocrine, and metabolic disorders are also described, however, the exact pathophysiologic mechanisms remain sufficiently understudied [7,8,9,10,11].

The immunological profile of post-COVID-19 syndrome and CFS/ME includes impairment in the cytokine profile and immunoglobulin concentration, T- and B-cell activation, and a decrease in the cytotoxicity of the natural killer cells [12]. The presence of various autoantibodies has been described, in particular, antinuclear, antiphospholipid, antiganglioside antibodies, and antibodies to neurotransmitters and their receptors, mainly to β-1 and β-2 adrenergic receptors and M3 and M4 acetylcholine receptors [13,14,15]. A recent study has shown the immunologic response to the neural tissue autoantigens in post-COVID-19 patients as well as serological similarities between this condition and antiphospholipid syndrome [13].

One of the possible causes of the multiple and widespread symptoms in post-COVID-19 and CFS patients is considered to be a small fiber neuropathy [16,17]. This polyneuropathy of autoimmune nature can lead to the development of dysautonomia, explaining the presence of autonomic manifestations, weakness, and cognitive impairment, along with cerebral perfusion dysfunction [18]. The evaluation of the peripheral nervous system dysfunction can lead to the objectification of diagnostic criteria of post-COVID-19 syndrome and CFS.

Hearing loss is a common manifestation in several systemic autoimmune diseases [19,20,21]. A primary autoimmune hearing loss is also described, with the involvement of several inner ear autoantigens (protein zero, tubulin, cochlin, inner ear supporting cell antigen, collagen type II, and, especially, heat shock protein 70) [22]. Considering the development of small fiber neuropathy in patients with CFS and post-COVID-19 syndrome, impairments of the auditory and vestibular functions are also possible and reviewed in the studies [23,24,25]. Taking into account that these complaints may be noted by patients themselves only when the auditory dysfunction is progressing to mild or severe extent, hearing and vestibular studies may be an important part of the clinical investigation. Considering the frequent complaints of memory loss and attention deficit in patients with post-COVID-19 and CFS, the assessment of hearing seems to be a study that may clarify the pathophysiological mechanisms of the disease and allow the performance of a differential diagnosis. Assessment of vestibular disorders in this group of patients is required primarily in order to evaluate orthostatic dysfunction, which occurs in about 70% of cases [26].

## 2. Clinical Case Description

### 2.1. Patient’s Anamnesis

The patient, 41 years old, female, had complaints of constant weakness and fatigue, which did not allow her to fully perform daily routines and professional activity. She noted episodes of pyrexia up to 38.5 °C as a response to emotional events and stresses (average body temperature 36.9–37.3 °C), accompanied by flu-like symptoms. She also noted orthostatic intolerance, nausea and vomiting, as well as sleep disturbance, attention difficulties, dizziness, and even falls in the dark and regular episodes of herpes virus infection. Those complaints intensified gradually and significantly after an episode of SARS-CoV-2 infection in 2020. Given the presence of constant fatigue, as well as immunological, autonomic, and neurocognitive symptoms, the patient asked for medical help, suspecting CFS and post-COVID-19 syndrome.

Such complaints as constant fatigue and stress-associated pyrexia with flu-like symptoms the patient noted from childhood, but previously, they were episodic and had a mild severity. After COVID-19 infection and measle revaccination in December 2020, the symptoms became persistent and pronounced, worsening the patient’s daily activities. Over the past two years, she noted insomnia with early awakenings that were provoked by alcohol intake, overwork, and emotional stress. The patient also noted the presence of unsteadiness at night with periodic falls. In addition, after the SARS-CoV-2 infection, she began to notice an autonomic dysregulation, manifesting with orthostatic intolerance and nausea and vomiting with an increase in systolic blood pressure above 110 mm Hg. Moreover, the recurring episodes of herpes virus infection over the last few years were evaluated. In childhood, she suffered chickenpox, mumps, frequently occurring otitis, tonsillitis, pharyngitis, as well as recurring episodes of herpes viral infection. In 2016, a papilloma of the mammary gland was evaluated. Also in 2016, she was diagnosed with hypothyroidism, and treatment with levothyroxine was prescribed. No other chronic diseases were evaluated.

### 2.2. Results of Clinical and Laboratory Examinations

DePaul Symptom Questionnaire (DSQ-2) [7]: CFS/ME

Immunologic studies: CMV-IgG+, VCA IgG+, EBNA IgG+.

ECG: no pathological signs revealed.

Ultrasonography of brachiocephalic vessels: signs of vertebrogenic influence at the extracranial level of the vertebral arteries.

Brain MRI: no pathology evaluated.

Complete blood count, blood chemistry test, urinalysis: no pathology evaluated.

Neurological consultation: Preserved cognitive functions. Cranial nerves without pathology. Muscle strength in the upper and lower extremities D = S, no pathology evaluated. Deep reflexes were symmetrical. Superficial and deep sensitivity was preserved. Stable in the Romberg position. No gait abnormalities.

### 2.3. Vestibular Examination

All standard neurological vestibular tests were performed with normal results.

### 2.4. Auditory Examination

**Figure 1:**
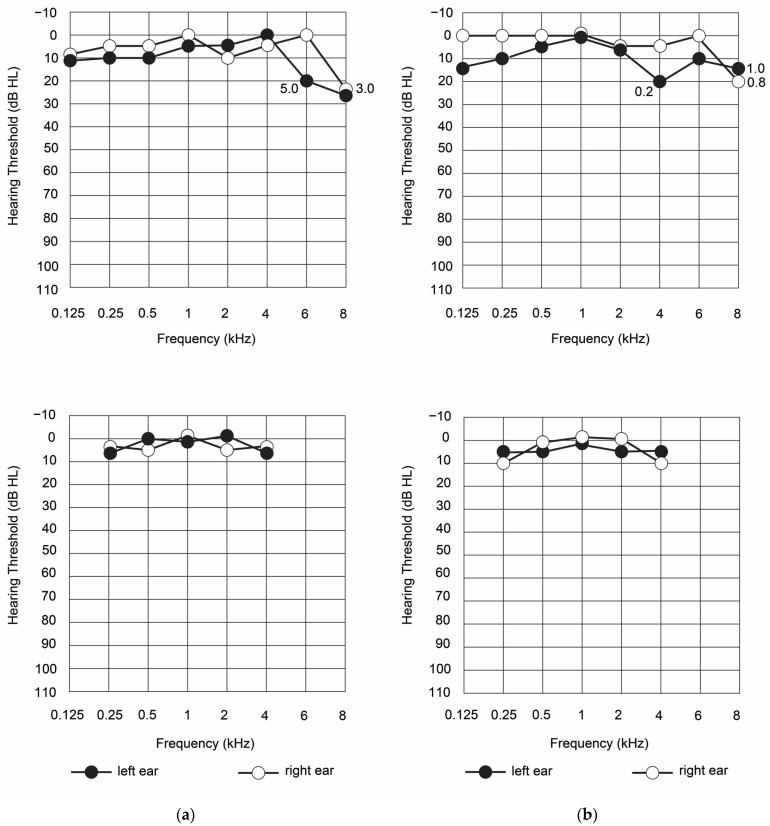
The results of air (**upper** panels) and bone (**bottom** panels) standard audiometry in the studied patient at the first appointment (**a**) and after 4 months of observation (**b**).

The results of air and bone standard audiometry showed that tonal hearing was also within the normal range (Figure 1).

A slight decrease in hearing loss up to 25 dB for both ears was found only at 8 kHz stimulation. The Luscher’s test was performed at frequencies of 6 and 8 kHz, and the intensity modulation thresholds for the left and right ears were 0.8 and 1.0 dB, respectively. Since the test result was negative, no dysfunction of the suprathreshold sound was detected. Thus, the patient’s peripheral hearing condition could be assessed as an initial pathological process in the organ of Corti.

The auditory processing disorder or central auditory processing disorder was tested using several tests. The binaural fusion test-audiometry with alternating binaural speech (BNR), which, according to different authors, is highly sensitive to the lesions of the brainstem [27] and to disorders of the higher-level auditory processing centers [28], was performed by the patient successfully. She recognized 100% of monosyllabic words presented in silence to the left and right ears, and 95% of such words in binaural listening when the first half of a word was delivered to one of the ears and the second was contralateral. The obtained result was normal. During the auditory memory examination using the pace auditory serial addition test (PASAT) [29], the patient added 55 out of 60 pairs of digits heard without errors, which is equivalent to 83% correct answers. This result corresponds to the average values shown by adult subjects from the healthy controls [29].

**Figure 2:**
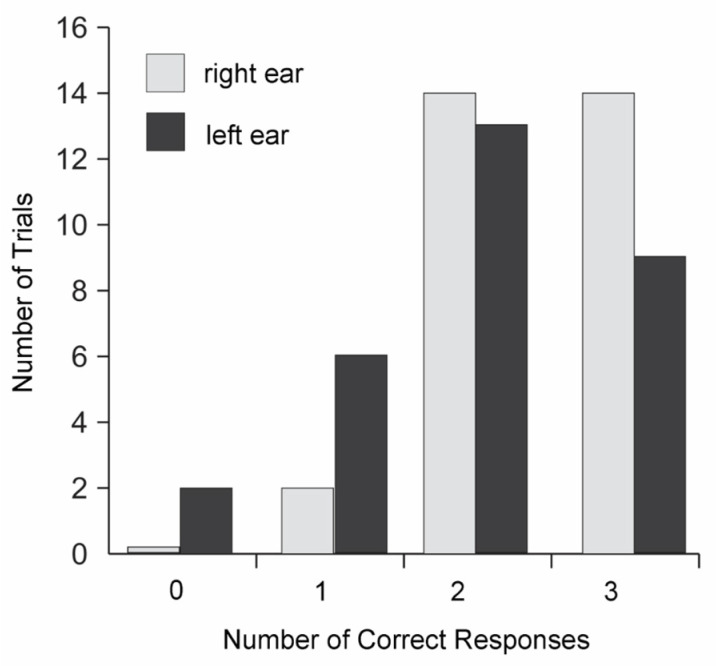
Dichotic test in the examined patient.

The other two central auditory processing tests the patient performed with significant difficulties. The dichotic word listening test, which detects dysfunction in the interhemispheric connections and changes in the functioning of the corpus callosum [30], was passed by the patient with a large number of errors. There were 66% correct answers from the left ear, and 81% from the right. The test revealed a significant predominance of the right ear. In the case of normal hearing, the number of correct answers in the dichotic test is not less than 90% [31]. The results of the dichotic test are shown in Figure 2. The patient was unable to reproduce all 6 words in any of the 30 auditions, and in 2 cases she did not reproduce any of the words presented to her left ear.

The gap detection test has been applied as a modified version [32]. The patient was unable to pass this test in any of the five series: neither with tone pulses, nor with click stimulation. Re-examination four months later confirmed this result. Noteworthy is that this test is sensitive to detecting cortical abnormalities, especially in the left hemisphere [33].

**Figure 3:**
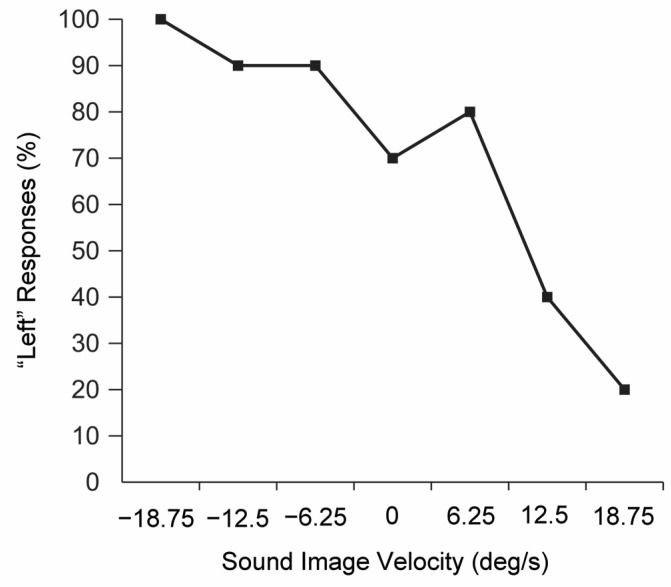
The test of localizing a sound image in the examined patient.

The test of localizing a sound image and the direction of its movement was performed correctly in up to 80% of cases for both directions of movement, to the right and to the left (Figure 3).

### 2.5. Small Fiber Neuropathy Evaluation

To study the possible presence of small fiber neuropathy in this patient, two tests were performed: a skin biopsy with the counting of the intraepidermal nerve fibers, and confocal microscopy of the cornea. The skin biopsy was taken from the lateral part of the thigh and prepared and fixed according to the standard protocol.

Intraepidermal small fiber density in the patient was 9.4 fibers per mm, when the 5th percentile normal value for her age is considered to be 5.7 fibers per mm [34]. Therefore, no signs of small fiber neuropathy in the distal part of the extremity were evaluated.

**Figure 4:**
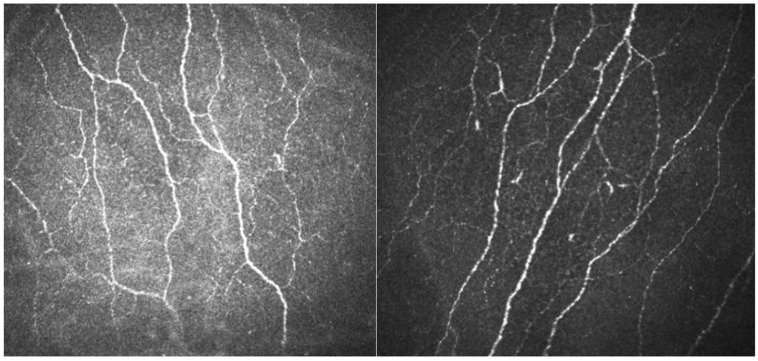
Corneal confocal microscopy with the assessment of the subbasal plexus.

To specify this result, corneal confocal microscopy was performed, where the images of the subbasal corneal plexus were obtained (Figure 4).

Semi-automatic analysis of the obtained images has shown the following results (Table 1).

According to the obtained results, density and length of the corneal nerve fibers in the patient were within normal values, though CNBD and CNFL were lower than gender- and age-corrected medians.

## 3. Discussion

Examination of the auditory function of the patient assessed both the peripheral and central parts of the auditory system. The peripheral hearing function was assessed by two tests: tone threshold audiometry and Luscher’s test. The patient’s tonal hearing thresholds did not exceed 20 dB at any audiometric frequencies, with the exception of 8 kHz. At this frequency, they remained to be 25 dB for the left and right ears, which shows initial pathological changes in the organ of Corti. The Luscher’s test, assessing the intensity modulation thresholds, was performed for those frequencies at which the patient’s hearing loss was 20 dB or more. The assessment of the intensity modulation thresholds at those frequencies showed that hearing thresholds were normal, therefore, the dynamic range of hearing remains intact [36]. No frequencies at which the patient’s hearing loss was 20 dB or more were evaluated, and there were no deviations in the intensity modulation perception thresholds from the normal values, showing normal cochlear compression mechanism. Thus, the results of the peripheral audiometric examination show the absence of pathological changes in the peripheral part of the auditory pathways in the patient; however, the data of the tonal threshold audiometry indicated the presence of initial degenerative changes in the hair cells.

Tests for central auditory impairment included a gap detection test, a dichotic test, PASAT, and the ability to localize a moving sound image. The random gap detection test was aimed to assess the auditory temporal resolution [37]. The neuronal structures involved in the detection of short pauses are concentrated in the left primary auditory cortex [38,39]. The test results showed that the patient had significant difficulties in detecting a pause for all types of signals: broadband clicks and tones (0.5, 1, 2 and 4 kHz).

The dichotic test made it possible to assess the state of speech hearing and identify the leading ear in the perception of speech information. According to the results of this test, the patient showed a pronounced preference for information coming to the right ear when perceiving speech stimuli, which indicates the main role of the left hemisphere in the analysis of verbal auditory information. The percentage of correctly recognized words applied to the left ear was 61%, which is significantly lower than the normal values [40]. This result may indicate dysfunction of the structures of the right hemisphere, in which the analysis of auditory verbal information takes place. The possibility of such dysfunction in patients with CFS is supported by the results of a study [41], which analyzed the degree of correlation (connection) between the activity of different parts of the cerebral cortex in individuals with CFS and healthy subjects at rest using the resting state fMRI. CFS patients showed a weaker association of the anterior cingulate cortex (ACC) with the right planum temporale (rPT), as well as with the right Heschl’s gyrus (rHG), than healthy volunteers. The functional significance of PT and HG in the auditory central processing is great and includes both the analysis of the fine temporal structure of sound stimuli [42] and the performance of tasks related to speech processing. ACC is a cortex area that is activated during the so-called “conflicted” tasks in which incentives can compete with each other [43]. Falkenber et al. [44] showed that dichotically-presented verbal signals can also act as stimuli that cause activation in the ACC. Thus, the difficulties we identified in the patient could be due to the weakening of the connection between the regions of the cortex responsible for perception (rPT and rHG) and subsequent analysis (ACC) of dichotically-presented speech stimuli.

Auditory-evoked potentials have not been performed due to the decision to apply different tests to assess central and peripheral auditory pathways (eg., binaural fusion test). This decision was made based on the following considerations. If a hearing impairment is detected, the use of standard methods involving short- and medium-latency auditory potentials is impractical, since the nature of auditory disorders clearly indicates a dysfunction of the auditory cortex. In this case, long-latent auditory potentials can be used, and, in their classical application, this method is ineffective because normally there is a high variability of characteristics, including latency. It is possible to use the negativity of misalignment in the variant when stimuli differing in duration are used as a deviant. This technique is experimental and, in our conditions, requires the preparation of software adapted for this patient, which is impractical. From an ethical point of view, the use of a long and uncomfortable examination for the patient is not justified by clinical practice, since the main clinical factors of the use of AEP in the diagnosis of CAPD (central hearing processing disorders) are the following:

- subjective (psychoacoustic) techniques did not allow clear identification of the nature of the dysfunction;
- the data of subjective methods are unreliable because they depend on the attention, motivation, and level of cognitive development of the listener;
- children’s age prevents the conduct of a full battery of psychoacoustic tests;
- there are neurological disorders that do not allow the performance of the battery of psychoacoustic tests;
- clarification of the localization of the disorder in the central parts of the auditory system is required if CAPD is detected on the basis of subjective techniques;
- the inability to conduct psychoacoustic tests in the patient’s native language.

Considering all these reasons, only subjective tests were used to assess the hearing state.

Note that speech audiometry tests have an obvious disadvantage due to the use of the patient’s cognitive resources for their successful implementation [45]. First of all, we are considering the participation of auditory memory and auditory attention. From this point of view, the use of speech tests makes it difficult to separate the actual auditory and cognitive problems of the patient. Therefore, the use of non-speech tests along with speech tests, such as the pause detection test or the sound lateralization test, allow us to clarify the nature of the dysfunction. Medium- and long-latent auditory potentials can provide significant assistance in assessing the state of auditory function in order to separate auditory and cognitive impairments. Depending on the level of localization of the alleged functional disorders, various variants of the AEP method may be used, the obvious advantage of which is its objectivity and independence from the patient’s level of proficiency in the language in which the examination is performed.

Thus, it is desirable to include tests for central hearing disorders in the screening examination of hearing in post-corneal syndrome. It follows from the above that the combination of one of the variants of the dichotic speech test and the test for the state of temporary auditory analysis may be sufficient. This approach, in particular, is confirmed by the results of [46], which used a combination of the dichotic digits test (DDT) and the gap in noise (GIN) detection test to assess age-related changes in CAPD. The advantage of psychoacoustic testing is accessibility, due to the relative simplicity and lack of additional equipment for the implementation of techniques. When identifying problems and the need for a deeper examination of the patient, it is desirable to use objective methods using medium- and long-latent potentials, which will require the development of unified protocols for such examinations and recommendations.

The alternate binaural speech test, which is a binaural interaction test, assesses the ability to perceive and integrate verbal information sequentially arriving at the left and right ears [47]. The patient successfully performed it, thus, the function of integration in time of the verbal information, sequentially arriving at the left and right ears, was preserved. This result indicates both the brainstem structure’s maintenance and the preservation of the auditory cortex on the left, responsible for the perception of speech stimuli, and is in good agreement with the results of the dichotic test, which showed a pronounced preference for the right ear (i.e., the left hemisphere). Apparently, it was the process of dividing competing signals and connecting short-term memory resources, which requires activation of areas of the cerebral cortex outside the primary auditory cortex, that determined the patient’s low results in the dichotic test as compared with the alternate binaural speech test.

The results of the PASAT test, which evaluated divided-attention/working memory processes, in the patient were within the normal range. It is known that patients with CFS, regardless of whether they have difficulty passing the PASAT test or not, often experience more extensive cortical activation during this test than in healthy subjects [48]. Patients have additional symmetric activation of the 24/32 (ACC) and 40 (supramarginal area) areas, as well as left-sided activation of the 45/47 area (inferior frontal gyrus). The authors of the study, who showed these differences, believe that such excessive neuronal activity may indicate a higher mental effort when performing complex auditory tasks that require concentration and memory resources, which leads to fatigue and subjective perception of such tasks as difficult ones. The pronounced fatigue of the patient after passing the test could indicate excessive activation of the cortex, typical for this disease.

Difficulty with hearing orientation is one of the important features of central disorders. Considering the fact that the auditory disorders of the patient were selective, we applied the test for the localization of moving sound images, which was passed by the patient successfully. The results indicated that the patient had no dysfunction in the auditory analysis of movement at the subcortical level.

The pathogenesis of hearing disorders in patients with post-COVID-19 syndrome and CFS/ME requires further investigation. Numerous studies on autoimmune hearing dysfunction show that the frequency of such disorders is very variable [49,50,51]. Autoantibodies to the structures of the inner ear can lead to cochlear fibrosis or ossification, after which even cochlear implantation will not show satisfactory results [52]. Recently, several studies have appeared covering post-COVID-19 hearing loss, however, according to our best knowledge, they all describe dysfunction in the peripheral part of the auditory system [53,54,55]. The causes may include a microcirculatory dysregulation in the inner ear, described in a number of case studies [53]. Most authors describe sensorineural hearing loss, partial or complete, and the positive effect of corticosteroids [54]. Some works emphasize the temporary nature of such disorders with subsequent recovery [55]. However, in our case study, despite the presence of cognitive and vestibular symptoms in a patient after a SARS-CoV-2 infection, no dysfunction of the peripheral nervous system was detected during profound testing of auditory and vestibular functions, as well as studies of small nerve fibers. However, a dysfunction of the primary auditory cortex may be suspected, a post-viral complication which has not been described in the literature before. To our best knowledge, there was only one study where vestibular disorders in patients with post-COVID-19 syndrome probably had a central genesis, but the small sample size did not allow the authors to have statistically significant results [56]. Subtle central nervous system dysfunction in patients with post-viral complications and no signs of lesions on the MRI studies may be an important issue explaining neurocognitive complaints in this group of patients and needs further research.

## 4. Conclusions

A detailed examination of the state of the patient’s peripheral and central hearing revealed a deficit in temporal auditory analysis and difficulties in processing speech information with competing signals. Both tests, which caused difficulties for the patient, showed a dysfunction of temporal auditory analysis (gap detection test and dichotic test). At the same time, no impairments to auditory memory and attention were found (PASAT). The function of localizing the sound source, the mechanisms of which primarily involve the brainstem structures of the ascending auditory tract, was also preserved. The selective nature of the impairment of auditory functions with normal tonal hearing indicates the central genesis of the identified disorders, which indirectly shows the possible localization of the deficit of auditory processing in the primary auditory cortex area on the right. This observation may also be supported by the absence of damage to the nervous system shown in skin biopsy and confocal microscopy of the cornea. The detected deterioration in the processing of temporal and verbal auditory information can be a significant factor contributing to additional overload of the neural activity and leading to chronic fatigue when performing daily activities in patients with CFS and post-COVID-19 complications.

## Figures and Tables

**Table 1:** The results of corneal confocal microscopy in the patient.

	CNFD, Corneal Nerve Fiber Density	CNBD, Corneal Nerve Branch Density	CNFL, Corneal Nerve Fiber Length
Patient’s results	29.86	49.30	18.79
Normal median values for 36–45 years, female [35]	28.56	63.27	23.28
Normal 5th quartile for 36–45 years, female [35]	14.79	18.19	12.48
